# Day-to-Day Variability of Postural Sway and Its Association With Cognitive Function in Older Adults: A Pilot Study

**DOI:** 10.3389/fnagi.2018.00126

**Published:** 2018-05-04

**Authors:** Julia M. Leach, Martina Mancini, Jeffrey A. Kaye, Tamara L. Hayes, Fay B. Horak

**Affiliations:** ^1^Personal Health Systems Laboratory, Department of Electrical, Electronic, and Information Engineering, University of Bologna, Bologna, Italy; ^2^Point of Care Laboratory, Department of Biomedical Engineering, Oregon Health & Science University, Portland, OR, United States; ^3^Oregon Center for Aging & Technology, Oregon Health & Science University, Portland, OR, United States; ^4^Balance Disorders Laboratory, Department of Neurology, Oregon Health & Science University, Portland, OR, United States; ^5^Layton Aging & Alzheimer’s Disease Center, Oregon Health & Science University, Portland, OR, United States; ^6^Portland Veterans Affairs Medical Center, Portland, OR, United States

**Keywords:** balance, cognitive decline, in-home monitoring, functional performance, motor control

## Abstract

**Introduction**: Increased variability in motor function has been observed during the initial stages of cognitive decline. However, the natural variability of postural control, as well as its association with cognitive status and decline, remains unknown. The objective of this pilot study was to characterize the day-to-day variability in postural sway in non-demented older adults. We hypothesized that older adults with a lower cognitive status would have higher day-to-day variability in postural sway.

**Materials and Methods**: A Nintendo Wii balance board (WBB) was used to quantify postural sway in the home twice daily for 30 days in 20 non-demented, community-dwelling older adults: once under a single-task condition and once under a dual-task condition (using a daily word search task administered via a Nook tablet). Mean sway distance, velocity, area, centroidal frequency and frequency dispersion were derived from the center of pressure data acquired from the WBB.

**Results**: Linear relationships were observed between the day-to-day variability in postural sway and cognitive status (indexed by cognitive global z-scores). More variability in time-domain postural sway (sway distance and area) and less variability in frequency-domain postural sway (centroidal sway frequency) were associated with a lower cognitive status under both the single- and dual-task conditions. Additionally, lower cognitive performance rates on the daily word search task were related to a lower cognitive status.

**Discussion**: This small pilot study conducted on a short time scale motivates large-scale implementations over more extended time periods. Tracking longitudinal changes in postural sway may further our understanding of early-stage postural decline and its association with cognitive decline and, in turn, may aid in the early detection of dementia during preclinical stages when the utility of disease-modifying therapies would be greatest.

## Introduction

The relationship between cognition and motor function has elicited significant interest since changes in motor function have been observed during the initial stages of cognitive decline (Aggarwal et al., 2006; Hayes et al., 2008; Buchman and Bennett, 2011). A marked increase in both cognitive and motor variability often occurs before the clinical manifestations of functional decline (MacDonald et al., 2006). Since changes in motor function have been shown to precede changes in cognition in elderly cohorts (Wang et al., 2006; Buracchio et al., 2010; Dodge et al., 2012), longitudinal monitoring of postural control, a specific motor function, may yield early detection of progressive motor decline and thus predict cognitive decline in our aging population (Kluger et al., 1997; MacDonald et al., 2003). Although cognitive dysfunction is associated with an increased fall risk (Buracchio et al., 2011), it is not known if postural control becomes more variable during the initial stages of cognitive decline.

Global cognition is often measured by brief cognitive screening tests, such as the Mini-Mental State Examination (MMSE; Folstein et al., 1975), which are relatively insensitive and subject to a ceiling effect unless moderate cognitive dysfunction is present (Trzepacz et al., 2015). Since this study was designed to target the initial stages of cognitive decline in a high-functioning, community-dwelling cohort, a more sensitive metric derived from extensive cognitive testing was desired. Hence, we quantified global cognition via the use of z-scores, a metric that represents an individual’s overall level of cognitive functioning or severity of cognitive impairment relative to the age-matched population mean. Cognitive status, indexed by the global z-score, was then analyzed against measures of postural control across time. If a change in the neural control of posture is associated with age-related changes in cognitive control, it is likely those postural and cognitive functions share common circuitry or mechanisms (Seidler et al., 2010; Park et al., 2016).

Postural control is a complex motor skill derived from the integration of dynamic sensorimotor processes and cognitive processing, an essential component of postural orientation and equilibrium (Horak, 2006). Postural instabilities arise with age (Horak et al., 1989; Peterka and Black, 1990; Maki and McIlroy, 1996; Woollacott, 2000) and are often exacerbated by impaired cognition (Verghese et al., 2002; Camicioli et al., 2007). Cognitively impaired older adults often experience more problems with balance and gait compared to their cognitively intact counterparts (Verghese et al., 2002; Camicioli et al., 2007). Measures of postural sway during quiet standing are often used to characterize postural control (Prieto et al., 1996) and have been shown to be sensitive to both mild and moderate-to-severe cognitive impairment (Leandri et al., 2009; Deschamps et al., 2014; Mignardot et al., 2014). Since an individual’s ability to control balance and gait while performing a cognitive task decreases with age and with cognitive impairment (Camicioli et al., 1997; Manckoundia et al., 2006; Granacher et al., 2011), measuring postural sway under a dual-task condition may enable the detection of the mild changes in postural control that occur during the initial stages of cognitive decline (Hayes et al., 2008; Buracchio et al., 2010; Dodge et al., 2012).

Despite our knowledge on the associations between cognitive and postural decline, we have a limited understanding of the interplay between these two systems and the time course of their decline since we currently do not acquire sensitive measures of both cognitive and postural control longitudinally. Postural control is assessed infrequently in both the clinical and research domains. Infrequent measures are fundamentally insufficient as they reflect one instance of performance, do not measure changes in performance and performance variability over time, and may mask true postural ability and decline (Kaye et al., 2011). Frequent, longitudinal postural sway measures would reveal the natural day-to-day variability patterns innate to the postural control system and would yield opportunity to detect changes in postural sway variability over time. To date, only one study has monitored postural sway daily, in the home, and for an extended period of time: McGrath et al. (2014) monitored 18 high-functioning, community-dwelling older adults for 8 weeks to establish baseline variability measures of postural sway in a healthy elderly cohort. The authors observed large inter- and intra-subject differences in posture that were not related to functional performance, suggesting that these variations represent natural movement variability instead of aberrant movement patterns (McGrath et al., 2014). Postural dual-tasking was not included in their protocol, but it would be an advantageous addition since the dual-task paradigm is often used to unmask subtle instabilities that would otherwise remain undetected.

In this study, we measured postural sway daily under both single- and dual-task conditions for 30 days in 20 non-demented, community-dwelling older adults over the age of 80. We specifically targeted an older cohort due to the increased sensitivity of the postural control system at advanced ages. Park et al. (2016) recently observed specific balance measures to decline more rapidly at old age, suggesting that a sample population over the age of 80 may exhibit distinct and potentially more susceptible postural control patterns. Our primary aim was to characterize the day-to-day variability in postural sway and determine whether there is a relationship between postural sway variability and cognitive status in non-demented older adults. This is the first study to acquire daily measures of postural sway under both a single- and a dual-task condition (i.e., standing while performing a cognitive task) and to assess the relationship between the day-to-day variability in postural sway and cognitive status in older adults. Since previous studies have observed increased variability in postural control during the initial stages of cognitive decline (Buracchio et al., 2010; Dodge et al., 2012), we hypothesized that non-demented older adults with a lower cognitive status would have more day-to-day variability in postural sway. In addition, we hypothesized that non-demented older adults with a lower cognitive status would perform worse on the daily cognitive task since a lower cognitive status likely implies inferior cognitive performance skills.

## Materials and Methods

### Subjects

#### Subject Characteristics

Twenty non-demented older adults enrolled in the Intelligent Systems for Assessing Aging Changes study (Kaye et al., 2011), a longitudinal community cohort study at the Oregon Health and Science University (OHSU) in Portland, OR, USA, were recruited for this pilot study. All subjects were ambulatory, community-dwelling older adults that met the following inclusion criteria: (1) nonfulfillment of the Diagnostic and Statistical Manual of Mental Disorders (DSM-IV) criteria for dementia (American Psychiatric Association, 2000); (2) preserved general cognitive functions as confirmed by a score of 24 or above on the MMSE (Folstein et al., 1975); (3) no significant impact on functional abilities, as confirmed by two or fewer activities marked as dependent on the Functional Assessment Questionnaire (FAQ; Pfeffer et al., 1982); (4) free of physical impairment that significantly inhibits stability; (5) no walking aid (i.e., walker or cane); (6) no known visual, vestibular, or somatosensory impairment greater than what is normal for one’s age as indicated by a standardized neurological examination and the near vision card testing; and (7) absence of significant depression as indicated by a score of less than 5 on the Geriatric Depression 15-item Scale (GDS; Sheikh and Yesavage, 1986).

The 20 subjects had a mean (standard deviation (SD)) age of 87.0 (6.8) years and 13 (65%) were women. All subjects were well educated (mean of 14.7 (2.3) years of education), generally healthy (mean Cumulative Illness Rating Scale (CIRS; Parmelee et al., 1995) score of 20.1 (2.3); mean MMSE score of 28.6 (2.0); and, mean GDS (Sheikh and Yesavage, 1986) score of 0.6 (0.7)), and had no known balance impairments (mean Tinetti Balance (Tinetti, 1986) score of 3.0 (3.6)).

#### Cognitive Status

Cognitive status was determined based on annual clinical and neuropsychological testing at OHSU’s NIA-Layton Aging and Alzheimer’s Disease Center (LAADC; detailed elsewhere in Kaye et al., 2011) and was defined by the subject’s global z-score. This global measure of cognition is a composite score derived from the integration of six different domain-specific z-scores, each representing specific cognitive abilities in six different cognitive domains. The six cognitive domains, as well as the two-to-four neuropsychological tests used to derive the z-score in each domain, are as follows: (1) *Memory*: WMS-R Logical Memory II Story A, WMS-R Visual Reproduction II and CERAD Word-List Recall; (2) *Language*: Boston Naming Test and category fluency (animals); (3) *Executive function*: letter fluency (CFL), Trail Making Test Part B and Stroop color-word conflict; (4) *Processing speed*: WAIS-R Digit Symbol, Trail Making Test Part A and Stroop color naming; (5) *Working memory*: WMS-R Digits Backward, WAIS-III Letter Number Sequencing or WAIS-IV Digit Sequencing and MMSE item WORLD backwards; and (6) *Visual perception/construction*: WAIS-R Block Design, WAIS-R Picture Completion and WMS-R Visual Reproduction I. All tests listed above are referenced elsewhere in Kaye et al. (2011).

**Figure 1:**
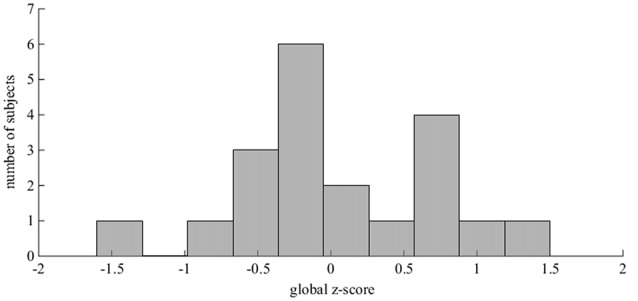
The distribution of the cognitive global z-scores. This histogram illustrates the spread of z-scores across the 20 subjects. These z-scores are based on normative data drawn from more than 3000 cognitively intact subjects and have been adjusted for age, sex and education (Kaye et al., 2011). The z-scores for 17 subjects fell within ± 1 standard deviation (SD) of the normative mean. Two subjects were relatively high functioning (1 < z-score < 1.5) and one subject was relatively low functioning (−1.5 < z-score < −1). This lower-functioning subject had a z-score of −1.44, lying just above the z-score cut-off for Mild cognitive impairment (MCI) according to the conventional Petersen/Winblad criteria, as operationalized by the Alzheimer’s Disease Neuroimaging Initiative (Petersen, 2004; Petersen and Morris, 2005).

The normative data used to derive the global z-scores were drawn from the first baseline evaluation of more than 3000 cognitively intact subjects from the LAADC (Kaye et al., 2011). The z-scores have been adjusted for age, sex and education. See Figure 1 for the distribution of global z-scores within this cohort. This cohort had a mean global z-score of 0.07 (0.70), meaning that although cognitive functioning abilities varied within this group of 20, all subjects were relatively high-functioning with no significant cognitive impairment.

### Experimental Procedures

The OHSU Institutional Review Board approved this study’s in-home technology setup and testing protocol (OHSU IRB #00008189) and all subjects gave informed written consent prior to participation.

#### In-Home Technology Setup and Testing Protocol

**Figure 2:**
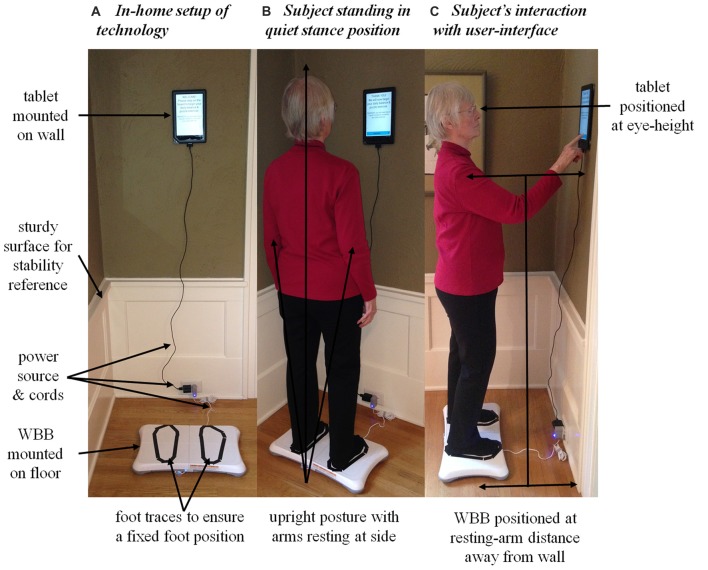
The in-home technology setup of the Nook tablet and the Nintendo Wii balance board (WBB) used to acquire daily center of pressure (CoP) postural sway measures.** (A)** The in-home technology setup: the WBB was mounted on the uncarpeted floor parallel to the wall and the tablet was mounted and leveled on the wall. The subject’s feet were traced with tape on the WBB’s usable surface to ensure a fixed foot position. Both devices were plugged into a power source to run continuously throughout the 30-day monitoring period. The system was positioned near a sturdy surface so the subject could grab hold and regain postural stability if need be. **(B)** The subject during quiet stance: maintaining natural upright posture with a fixed foot position (without shoes), arms resting at side, and a straight-ahead gaze. **(C)** The subject interacting with the user-interface. Note the position of the WBB and tablet relative to the wall and subject: the WBB was positioned at the subject’s resting-arm’s distance away from the wall to ensure a comfortable reach when interacting with the tablet; the tablet was centered relative to the WBB and positioned on the wall at the subject’s eye-height to ensure straight-ahead gaze. Written informed consent was obtained from the subject for the publication of these images.

A Nook tablet (Barnes and Noble, Inc., New York, NY, USA) and Wii balance board (WBB; Nintendo Co., Kyoto, Japan) were integrated into the current in-home technology remote sensing platform at the Oregon Center for Aging and Technology (ORCATECH; detailed elsewhere in Kaye et al., 2011) to monitor postural sway daily in the home. The WBB, an appropriate alternative to the “gold standard” force platform in situations where lower accuracy and precision is acceptable (Leach et al., 2014), was used to quantify postural sway via the displacement of the subject’s center of pressure (CoP) projected on the WBB’s usable surface. The tablet was used to: (1) acquire CoP data from the WBB via a Bluetooth connection; (2) administer instructions for the subject’s daily routine via a custom-written application; (3) store information input by the subject during the daily routine; and (4) transmit all posture and cognitive data to the ORCATECH data repository via a wireless internet connection. The in-home technology setup is illustrated in Figure 2.

**Figure 3:**
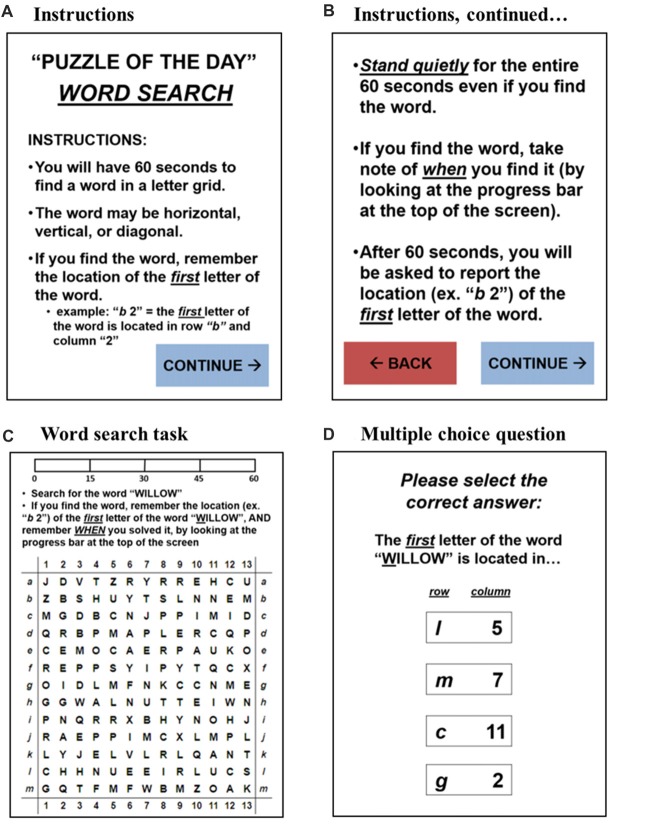
A mock-up of the user-interface for the dual-task condition: **(A,B)** the detailed instructions for the dual-task condition: the subject was able to toggle back and forth to ensure clarity on the instructions; the subject had to press “CONTINUE” to begin the daily word search task, ensuring he/she was ready to begin. **(C)** The word search task: the subject had to search for a specified word in a 13 × 13 letter grid; the subject was to note and remember the location of the first letter of the word upon finding the word in the letter grid; the progress bar at the top of the screen tracked time for the 60-s dual-task trial. **(D)** The multiple-choice question: after 60 s passed, the subject was prompted to report the solution to the word search task; the subject was instructed to simply guess if unsure.

Postural sway was measured twice daily: once under the single-task condition (i.e., the primary task of quiet standing) and again under the dual-task condition (i.e., quiet standing while performing the secondary word search task). The daily routine administered to measure postural sway took each subject about 3 min to complete. The custom-written application ran continuously on the tablet and responded when the subject stood on the WBB (Figures 2A,B). Once in quiet stance position, the subject pressed “CONTINUE” and the user-interface then guided the subject through the daily routine (Figure 2C). First, the 30-s single-task trial was administered to acquire daily baseline measures of postural sway. Then, the daily word search task was introduced as the cognitive load during the dual-task condition. The user-interface provided the subject with detailed instructions on how to complete the daily word search task while standing quietly. The subject pressed “CONTINUE” when ready to proceed with the 60-s dual-task trial. Upon completion, the subject was prompted to report the solution to the word search by answering a multiple-choice question. A mock-up of the user-interface for the dual-task condition is illustrated in Figure 3. A progress bar located at the top of the tablet’s screen was used to track time during both the 30-s single-task and 60-s dual-task trials.

### Data Analysis

All data was analyzed using MATLAB R2015a (MathWorks, Natick, MA, USA).

#### CoP Signals

The CoP signals were sampled at the WBB’s inconsistent rate of approximately 30 Hz and were resampled at 10 Hz during post-processing using the data averaging method detailed in Leach et al. (2014). All CoP signals (both the 30-s single-task and 60-s dual-task) were trimmed to a length of 25-s. The first 5 s were excluded from all signals to allow the subject time to settle into a true quiet stance position (Prieto et al., 1996). The first half of the dual-task CoP signals was used (as opposed to the second half) to increase the probability of quantifying postural sway under the dual-task condition. If the subject solved the word search before the 60-s allotment was over, his/her postural sway during the end of the dual-task trial would likely be similar to a single-task condition since he/she would no longer be working to solve the word search.

#### Postural Sway Measures and Postural Dual-Task Costs

Five postural sway measures were derived from the 25-s CoP signals to quantify both single- and dual-task postural sway daily. Four of the five measures were selected to represent distinct and independent features of the postural sway signal: mean sway distance (*MD*), mean sway velocity (*MV*), centroidal sway frequency (*fC*) and frequency dispersion (*FD*) (Maurer and Peterka, 2004; Rocchi et al., 2004). Sway area (*AREA*) was included to model the stabilogram and can be conceptualized as the product of *MD* and *MV* (Prieto et al., 1996).

Postural dual-task costs were computed for each postural sway measure to quantify the effect of the word search task on postural sway each day [dual-task cost = 100*(dual-task condition − single-task condition)/single-task condition]. The monthly means and day-to-day variability (quantified by variance) were computed for each postural sway measure and dual-task cost across the 30 days.

#### Cognitive Performance Measure

Cognitive performance was quantified by the subject’s performance on the daily word search task. Daily performance was reported as “correct” [1] or “incorrect” [0]. Cognitive performance rate (in percent correct) was acquired by averaging daily performance across the days in which the subject completed his/her daily routine.

### Statistical Analysis

Repeated measures analysis of variances (ANOVAs) were used to assess whether there was a difference in postural sway means or day-to-day variability under the single- vs. dual-task conditions and all assumptions were checked. An alpha of *p* < 0.05 was set as the threshold for statistical significance. Linear regression analyses were used to assess whether: (1) the monthly means or day-to-day variability in postural sway under both the single- and dual-task conditions were related to cognitive status; (2) the monthly means or day-to-day variability in postural dual-task cost were related to cognitive status; and (3) cognitive performance rate on the daily word search task during quiet standing was related to cognitive status. The least squares method was used for all linear regression analyses performed in this study.

## Results

### Protocol Adherence

On average, 3 days-worth of posture and cognitive data were missing across the 30 days for each subject. Subject adherence was not related to cognitive status.

### Postural Sway and Postural Dual-Task Cost Across Time

Postural sway measures were not different between the single- and dual-task conditions: the word search task neither affected the monthly means nor the day-to-day variability in the five postural sway measures. The monthly means and day-to-day variability in single-task postural sway, dual-task postural sway and postural dual-task costs are reported in Table 1.

**Table 1:** The monthly means and day-to-day variability in postural sway and postural dual-task cost across 30 days.

	Measure	Units	A. Single-task Group mean ± SE	B. Dual-task Group mean ± SE	C. Dual-task cost (in %) Group mean ± SE
**I. Monthly means**	*MD*	*mm*	4.21 ± 0.31	3.68 ± 0.27	−8.81 ± 4.01
	*MV*	*mm/s*	15.29 ± 1.35	14.33 ± 1.41	−6.78 ± 1.62
	*AREA*	*mm^2^*/s	21.71 ± 3.17	17.79 ± 2.48	−10.34 ± 4.92
	*fC*	*Hz*	1.09 ± 0.06	1.18 ± 0.68	11.60 ± 3.28
	*FD*	—	0.76 ± 0.01	0.76 ± 0.01	−0.03 ± 0.80
**II. Day-to-day variability**	*MD*	*mm^2^*	0.85 ± 0.15	0.75 ± 0.16	680.80 ± 128.85
	*MV*	*mm^2^*/s^2^	7.14 ± 2.75	6.51 ± 2.38	177.03 ± 27.97
	*AREA*	*mm^4^*/s^2^	73.42 ± 19.16	48.66 ± 14.78	1419.26 ± 238.03
	*fC*	*Hz^2^*	0.04 ± 0.01	0.05 ± 0.01	723.57 ± 82.95
	*FD*	—	0.00 ± 0.00	0.00 ± 0.00	59.92 ± 4.06

### Postural Sway Variability and Cognitive Status

**Figure 4:**
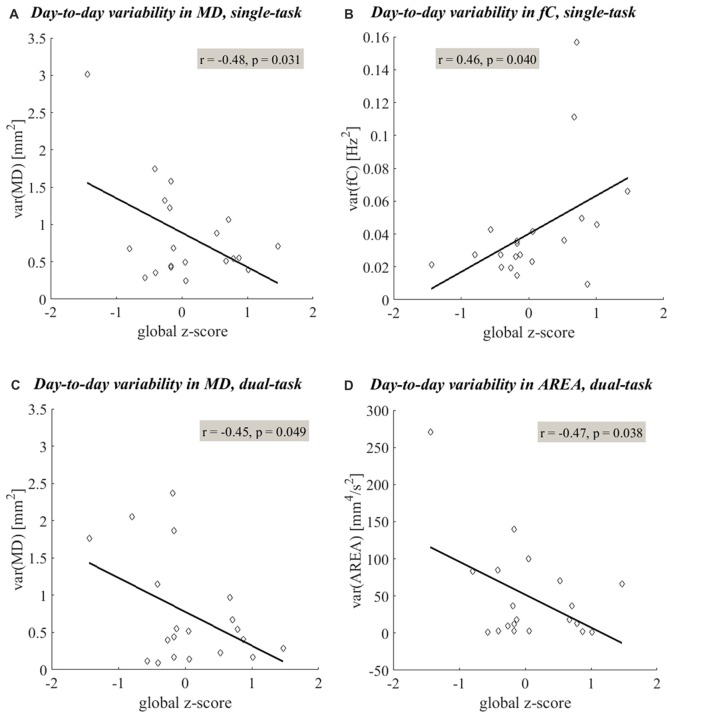
Linear relationships between the day-to-day variability in postural sway and cognitive status. Linear regression shows significant linear relationships (*p* < 0.05) between the day-to-day variability in postural sway measures and global z-scores. More variability in time-domain postural sway (quantified by *MD* **(A,C)** and *AREA* **(D)**) and less variability in frequency-domain postural sway (quantified by *fC* **(B)**) were related to lower global z-scores. The linear trends observed under the single- and dual-task conditions are shown in plots **(A–D)**, respectively.

**Figure 5:**
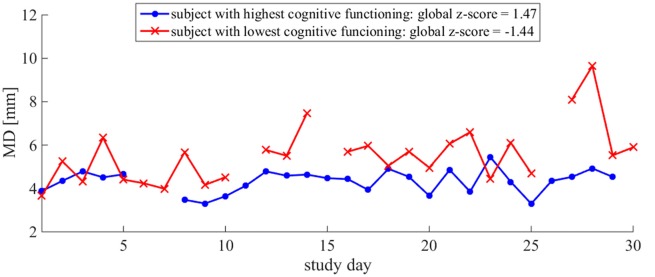
*MD* time series illustrates the difference in day-to-day variability between relative high and low cognitive statuses: more variability in *MD* is observed in the older adult with the lower global z-score. Daily *MD* measures for two subjects are plotted. The subject with the lowest global z-score is plotted in red and the subject with the highest global z-score is plotted in blue. The lines are discontinuous due to missing data on some days. Both subjects had 3 days of missing data over the course of the 30-day monitoring period.

The day-to-day variability in both single- and dual-task postural sway was significantly related to cognitive status. More day-to-day variability in time-domain postural sway and less day-to-day variability in frequency-domain postural sway were related to lower global z-scores. Under the single-task condition, more variability in *MD* and less variability in *fC* were related to lower global z-scores (Table 2, **(II.A)**, Figures 4A,B). Under the dual-task condition, more variability in both *MD* and *AREA* was related to lower global z-scores (Table 2, **(II.B)**, Figures 4C,D, 5). The day-to-day variability in postural dual-task cost (Table 2, **(II.C)**) and the monthly means in single-task postural sway, dual-task postural sway, and postural dual-task cost (Table 2, **(I.A–C)**) were not related to cognitive status.

**Table 2:** Linear relationships between the monthly means and day-to-day variability in postural sway with cognitive status (global z-scores).

	Measure	A. Single-task			B. Dual-task			C. Dual-task cost		
		*r*	*F*	*p*	*r*	*F*	*p*	*r*	*F*	*p*
**I. Monthly means**	*MD*	−0.34	2.41	0.138	−0.32	2.11	0.163	0.02	0.01	0.931
	*MV*	0.04	0.03	0.870	0.06	0.07	0.799	−0.05	0.05	0.824
	*AREA*	−0.27	0.55	0.468	−0.18	0.57	0.460	−0.06	0.06	0.813
	*fC*	0.43	4.17	0.056	0.29	1.59	0.223	−0.18	0.58	0.457
	*FD*	−0.05	0.05	0.827	−0.12	0.24	0.629	−0.12	0.24	0.631
**II. Day-to-day variability**	*MD*	**−0.48**	**5.51**	**0.031**	**−0.45**	**4.47**	**0.049**	0.08	0.13	0.723
	*MV*	0.18	0.61	0.446	0.16	0.50	0.491	−0.17	0.51	0.485
	*AREA*	−0.25	1.15	0.297	**−0.47**	**5.03**	**0.038**	−0.06	0.06	0.804
	*fC*	**0.46**	**4.91**	**0.040**	0.26	1.33	0.265	−0.27	1.40	0.252
	*FD*	0.21	0.84	0.371	0.05	0.04	0.842	0.00	0.00	0.992

*Results from the linear regression analyzing relationships between the monthly means **(I)** and day-to-day variability **(II)** in single-task postural sway **(A)**, dual-task postural sway **(B)**, and postural dual-task cost **(C)** and cognitive status (indexed by global z-scores). Significant linear relationships were observed between the day-to-day variability in both single- **(II.A)** and dual-task **(II.B)** postural sway and global z-scores. There were no relationships between the day-to-day variability in postural dual-task cost **(II.C)** and global z-scores, nor were there relationships between the monthly means of single-task postural sway **(I.A)**, dual-task postural sway **(I.B)**, or postural dual-task cost **(I.C)** and global z-scores. Bolded values represent *p* < 0.05*.

### Cognitive Performance Rates and Cognitive Status

**Figure 6:**
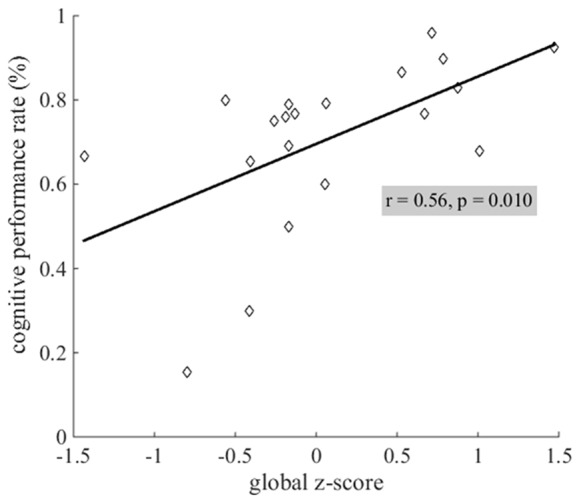
Linear relationship between cognitive performance rates and cognitive status. Lower performance rates (in %) on the daily word search task were related to lower global z-scores.

Cognitive performance rate on the daily word search task was significantly related to cognitive status: lower performance rates were related to lower global z-scores (Figure 6).

## Discussion

### In-Home Monitoring of Postural Sway Is Feasible in Older Adults

In-home monitoring of postural sway both with and without cognitive dual-tasking was feasible in our older adult population. All subjects adhered well to the testing protocol, regardless of cognitive status. This elderly cohort was generally healthy, well educated, and had experience with longitudinal, in-home, technology-driven studies. Future studies will need to be conducted with larger, more diverse sample populations to establish the generalizability of this approach.

### Day-to-Day Variability in Postural Sway Is Associated With a Lower Cognitive Status

More day-to-day variability in postural sway distance and area was related to a lower cognitive status (Table 2, Figures 4, 5), supporting our primary hypothesis. This finding is consistent with the literature and couples well with the results from a previous ORCATECH study in which Dodge et al. (2012) observed associations between week-to-week gait speed variability and cognitive decline. Gait speed variability trajectories across 182 weeks were associated with the severity of mild cognitive impairment (MCI): an initial period of increased variability followed by an accelerated decrease in variability was associated with early-stage MCI, whereas a sustained and gradual decrease in variability was associated with late-stage MCI. Although we did not expect to see meaningful changes in postural sway variability over time since our monitoring period was much shorter (4 vs. 182 weeks), we did predict postural sway variability to relate to cognitive status since gait speed variability was sensitive to early-stage MCI. Our finding supports our hypothesis and suggests that postural sway variability may also be sensitive to cognitive status and decline.

The consistencies between the Dodge et al. (2012) study and this study suggest that some subjects within our cohort of 20 may have preclinical or early-stage MCI. According to the theory on declining biologic systems (MacDonald et al., 2006), instead of abrupt failure, the affected system often demonstrates an initial period of increased variability before functional decline (as Dodge et al., 2012 observed in early-stage MCI). This is likely the result of physiologic or functional reserve attempting to compensate for the disease-related dysfunction. However, once compensatory systems fail or pathologic burdens exceed the level sustainable by reserve, the period of increased variability dissipates and functional decline occurs (as Dodge et al., 2012 observed in late-stage MCI). In our study, the relationship between postural sway variability and cognitive status was observed in the absence of clinical impairment of balance (mean Tinetti Balance score of 3.0 (3.6)) and cognition (MMSE score ≥ 24). Thus, the subjects with a lower cognitive status within this cohort likely still have sufficient functional reserve to prevent debilitating postural or cognitive disability.

Increased variability of posture or gait is thought to represent less automatic (more cortically controlled) function (Yogev-Seligmann et al., 2008; Hausdorff and Buchman, 2013). Furthermore, objective measures of postural sway, in conjunction with gait measures, provide non-redundant information about functional mobility (Mancini et al., 2012; Horak et al., 2016). Like gait speed, postural sway distance and area are time-domain measures of postural control. Although measures of postural sway and gait are both used to characterize functional mobility, the neural circuits involved in maintaining upright posture during quiet standing are independent from those required for walking (Horak et al., 2016) and are thought to require more high-level cognitive processing (Montero-Odasso et al., 2012; Takakusaki, 2013). Therefore, postural sway distance and area characterize distinct features of functional mobility and may enable a sensitive analysis of motor decline during the preclinical stages of MCI and dementia.

In contrast with our results for time-domain postural sway, less day-to-day variability in postural sway frequency was related to a lower cognitive status (Table 2, Figure 4B). In a recent 2016 study, Horak et al. (2016) found that sway frequency and distance/area represent two distinct features of functional mobility. Our findings agree that the frequency-domain of postural sway may be influenced independently from the time-domain during the initial stages of decline.

### Lower Cognitive Performance, but Not Postural Dual-Task Cost, Is Associated With a Lower Cognitive Status

Lower cognitive performance rates during the dual-task condition were related to a lower cognitive status, supporting our secondary hypothesis (Figure 6). The older adults who tested lower in global cognition during the standardized psychometric testing specified in Kaye et al. (2011) performed worse on our daily word search task. This suggests we designed a cognitive dual-task sensitive to global cognition. However, since the word search task is not a classic neuropsychological test, the cognitive skills necessary to accomplish the word search, as well as the sensitivity of the task itself, has not been validated.

Although the cognitive task’s measure of cognition was as anticipated, the cognitive task’s effect on postural sway was not. In fact, we predicted the cognitive task to increase postural sway and to have a greater effect on postural sway in older adults with a lower cognitive status. However, no differences in postural sway were apparent between the single- and dual-task conditions and the postural dual-task cost was not related to cognitive status. These results may be explained by the type of cognitive dual-task used in this study.

Cognitive task selection was both driven and limited by our effort to acquire sound measures of postural sway in older adults with daily testing in the home. We selected a secondary cognitive task of reasonable difficulty level to sufficiently draw attention away from the primary postural task. We needed a cognitive task without a verbal or physical response since both would affect the CoP signal. Articulation provokes changes in the respiratory pattern which increases postural sway frequency (Dault et al., 2003) and a physical movement, such as lifting an arm to touch the tablet, would increase postural sway as well. Therefore, the cognitive task in this pilot study was restricted to mental and visual components in order to maintain the integrity of the CoP signal. Although most cognitive tasks increase postural sway, some nonverbal mental (Andersson et al., 2002; Yardley et al., 2002) and visual search (Huxhold et al., 2006; Prado et al., 2007) tasks have been shown to decrease postural sway in older adults. Similarly, we observed a decrease in postural sway during the word search task (negative postural dual-task costs for the time-domain measures in Table 1, (**I.C**)). However, this observed decrease lacks statistical significance (as reported in “Postural Sway and Postural Dual-Task Cost Across Time” section). We posit that the constantly moving object within the subjects’ field of vision (i.e., the progress bar at the top of the tablet’s screen during both the single- and dual-task trials, as illustrated in Figure 2) may explain why there was not a significant difference in postural sway between the single- and dual-task trials. Since visual tracking is a cognitive task that can reduce postural sway (Huxhold et al., 2006), it is possible that our single-task trial was not truly a single task, thus dampening the postural dual-task costs.

Since we found the day-to-day variability in postural sway to be associated with cognitive status under both the single- and dual-task condition, and since there was no effect of cognitive status on postural dual-task cost, an added cognitive load may not be necessary to observe the differences in postural sway variability between cognitively intact and mildly cognitively impaired older adults. Removing the cognitive dual-task simplifies the in-home testing protocol by reducing the amount of time and resources necessary for in-home monitoring of postural sway. But before altering the experimental setup and removing the cognitive dual-task entirely, one must first confirm that the significant findings from this pilot study can be reproduced in a situation where the single-task condition is truly a single task (i.e., quiet stance without the potential of visual tracking).

### Study Limitations and Future Directions

The small size of our sample population is an obvious limitation of this pilot study. Since our small cohort limited our statistical power, we did not correct for multiple comparisons in our statistical analysis. We also cannot make strong inferences regarding outliers in our dataset. The two subjects out of 20 with the lowest global z-score (both below one SD of the group’s mean (0.07 (0.70))) happen to serve as the potential outliers in both Figures 4A,D, 6. We are hesitant to consider these subjects as true outliers though since increased variability in postural control has been observed during the initial stages of cognitive decline (Dodge et al., 2012). Because both the cognitive and postural control systems have been shown to demonstrate an initial period of increased variability during the depreciation of physiologic reserve (MacDonald et al., 2006), and because the clinical manifestations of postural decline have been shown to precede that of cognitive decline (Buracchio et al., 2010), the postural profile expressed by these subjects may in fact be indicative of true functional state.

The advanced age, preserved cognition and intact postural control of our cohort serve as additional limitations to this pilot study. Our group of 20 subjects represents a superior sample, especially for their age. Since a recent study observed specific balance measures to decline more rapidly at old-old age (Park et al., 2016), our findings may be specific to, or distinct from, other old age groups (e.g., ages 65–80). It would be interesting to assess the effect of age on variability patterns. Hence, extending this research to a larger sample population with a more diverse spread of age and cognitive and postural abilities would advance our knowledge on the interplay between age, cognition, postural control, and performance variability across time. For example, including subjects with more severe cognitive impairments (e.g., mid- to late-stage MCI and dementia) would enable us to determine whether increased variability in time-domain postural sway is, in fact, an early marker of cognitive decline. Additionally, following subjects for a longer period of time would expand our capacity to make inferences regarding the time-course trajectory of postural sway variability during cognitive decline.

This small pilot study conducted on a short time scale motivates large-scale implementations over more extended time periods. Tracking longitudinal changes in postural sway may further our understanding of early-stage postural decline and its association with cognitive decline and, in turn, may aid in the early detection of dementia during preclinical stages when the utility of disease-modifying therapies would be greatest.

## Author Contributions

JML was responsible for all activities associated with this project; co-planned the study design; obtained ethics approval; secured funding for all expenses relating to the project (technology and equipment, subject compensation, travel expenses, etc.); designed, tested, implemented and maintained the in-home technology setup; recruited, consented and trained subjects for in-home testing; monitored and managed data acquisition, transfer and storage; processed, analyzed and interpreted the data; and, prepared, revised and submitted the manuscript. MM contributed to the study design, data analysis and interpretation and manuscript preparation and revisions. JAK contributed to the study design, assisted with data interpretation and made revisions to the manuscript. TLH contributed to the initial stages of study design, technology acquisition and system development and testing. As principal investigator, FBH co-planned the study design, contributed to data analysis and interpretation, contributed to manuscript preparation and revisions and made the final decision to submit the manuscript for publication.

## Conflict of Interest Statement

The authors declare that the research was conducted in the absence of any commercial or financial relationships that could be construed as a potential conflict of interest.
